# Supplementary material for: Expanded vacuum-stable gels for multiplexed high-resolution spatial histopathology
Source: Nat Commun. 2023 Jul 7;14:4013. doi: 10.1038/s41467-023-39616-w (PMC10329015; doi:10.1038/s41467-023-39616-w)
Supplement: Supplementary file 1 — Supplementary Information document - FINAL [file 41467_2023_39616_MOESM1_ESM.pdf]

Supplementary Information for

Expanded Vacuum-Stable Gels for Multiplexed High Resolution Spatial Histopathology

Yunhao Bai, Bokai Zhu, John-Paul Oliveria, Bryan J. Cannon, Dorien Feyaerts, Marc Bosse, Kausalia Vijayaragavan, Noah F. Greenwald, Darci Phillips, Christian M. Schürch, Samuel M. Naik, Edward A. Ganio, Brice Gaudilliere, Scott J. Rodig, Michael B. Miller, Michael Angelo, Sean C. Bendall, Xavier Rovira-Clavé*†, Garry P. Nolan*†, Sizun Jiang*†

†These authors jointly supervised this work.

* Corresponding author(s). E-mail(s): x.rovira.c@gmail.com; gpnolan@stanford.edu; sjiang3@bidmc.harvard.edu;

This PDF file includes:

Supplementary Figures 1 to 12

Supplementary Notes 1

Supplementary Tables 1 to 10

Supplementary Figures

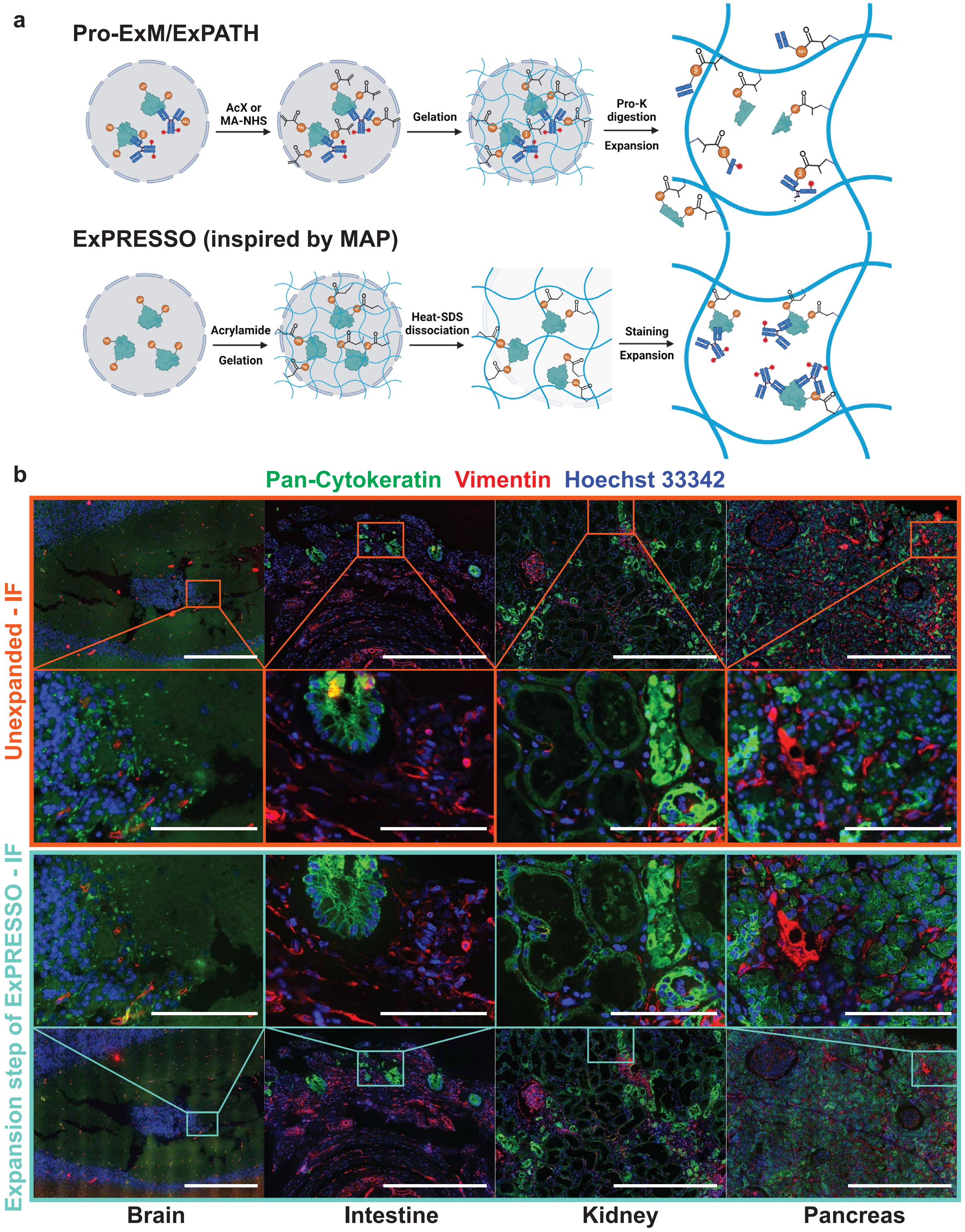

Supplementary Figure 1: Validation of the ExPRESSO workflow, related to Figure 1. **(a)** Schematic representation of two expansion approaches for protein detection. Top: The pro-ExM and ExPath frameworks first stain targeted proteins with antibodies, and then use a reagent (e.g., acryloyl-X or AcX) to anchor sample proteins and antibodies to the *in situ* polymerized gel network. Samples are then proteolytically digested (e.g., Proteinase K or Pro-K) and expanded. Bottom: The ExPRESSO process, inspired by MAP, first anchors sample proteins to an *in situ* polymerized gel network using a high concentration of acrylamide and then dissociates biomolecular complexes in the sample by a heat and SDS co-treatment. Samples are then stained with antibodies and expanded. **(b)** Representative fluorescence images of (left to right) brain, intestine, kidney, and pancreas human tumor sections before (top) and after (bottom) the expansion step of ExPRESSO. Samples were stained with vimentin, pan-Cytokeratin, and Hoechst 33342. The same regions on each tissue were imaged before and after expansion. Enlarged views from each box are shown. Scale bars (the expanded image scale bars indicate the pre-expansion dimensions based on a 3.7-fold expansion): 500 µm (top), 100 µm (top; enlarged), 500 µm (bottom), 100 µm (bottom; enlarged).

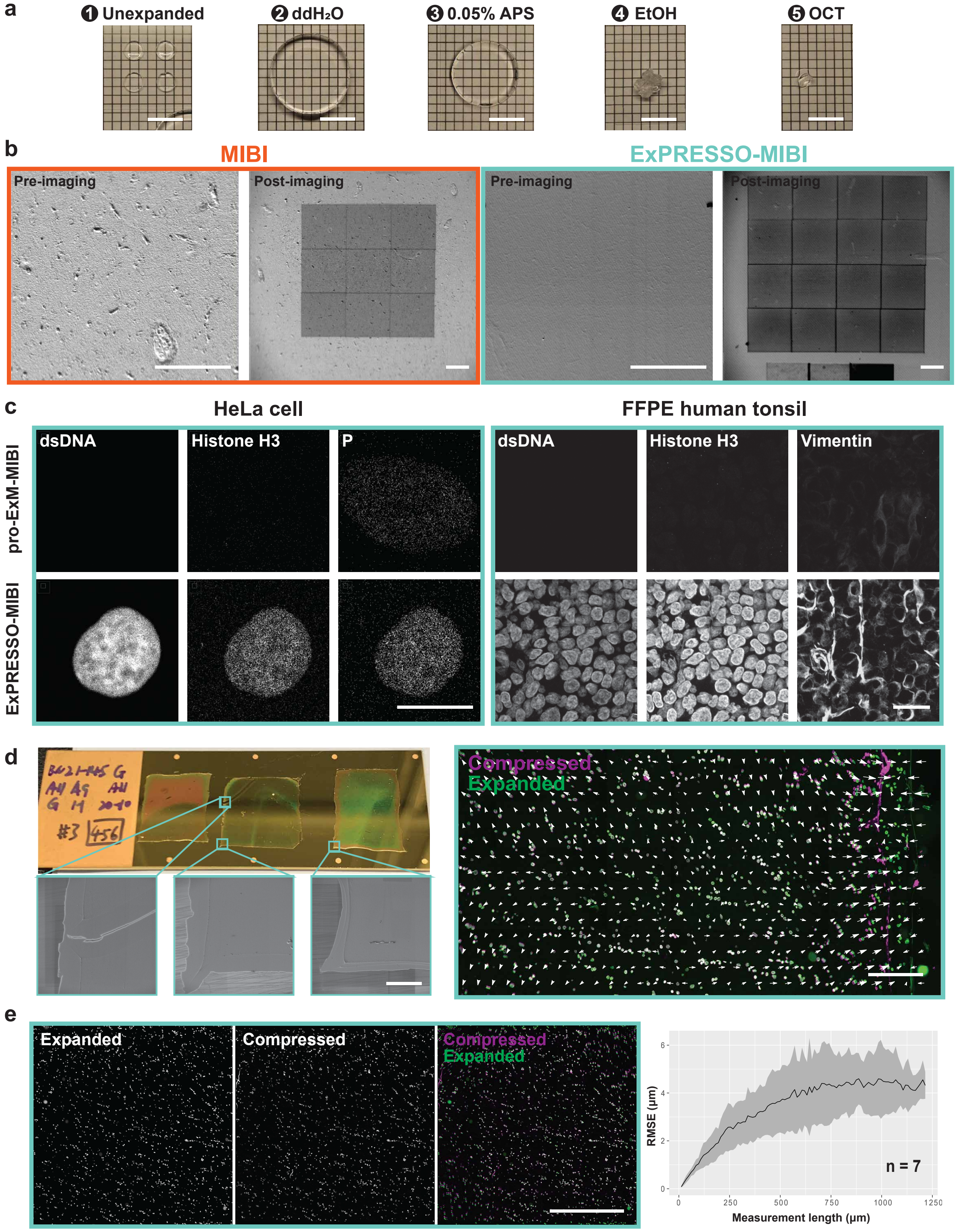

Supplementary Figure 2: Validation of the ExPRESSO workflow, related to Figure 1. (a) Images of ExPRESSO gels made with gaskets of 6 mm diameter are shown (1) unexpanded, (2) fully expanded in ddH$_2$O, (3) fully expanded then infused with 0.05% Ammonium persulfate (APS), (4) after soaking in neat EtOH, and (5) after infusion with OCT. Grid size: 2.5 mm, scale bars (all scale bars indicate physical measurement): 10 mm. **(b)** Representative secondary electron images of an unexpanded tissue (left) and an ExPRESSO-MIBI gel (right) before and after MIBI imaging. Scale bars (all scale bars indicate physical measurement): 200 µm. **(c)** Comparison of proExM (top) and ExPRESSO (bottom) processed HeLa cells (left) and Human FFPE tonsil tissue (right) with MIBI imaging. For the same channel in the different treatments, the counts are scaled to the same level. Scale bars (all scale bars indicate physical measurement): 50 µm (both). **(d)** Left: Representative ExPRESSO samples collapsed on gold slides. Slide size: 25 x 75 mm. Enlarged regions show that the distortions are predominantly limited to boundaries/edges after compression. Right: An overlay of the same region of an Human brain FFPE Gyrus section pre- (Green) and post-compression (Magenta), with vector field plot overlaid as white arrows, shows the distortions are generally limited to the dried out edge boundaries. Scale bar (physical size): 500 µm (both). **(e)** Left: representative view of Hoechst 33342 in stained, expanded, and compressed brain sections used for distortion error calculation. The size of each region is 4.97 x 4.97 mm (physical size). Right: Plot showing the root mean square error (RMSE, n = 7) with plus or minus standard deviation between pre-expansion and post-expansion nuclear channel above at different lengths (back calculated based on a 3.7-fold expansion). Scale bar (indicates the pre-expansion dimensions based on a 3.7-fold expansion): 500 µm.

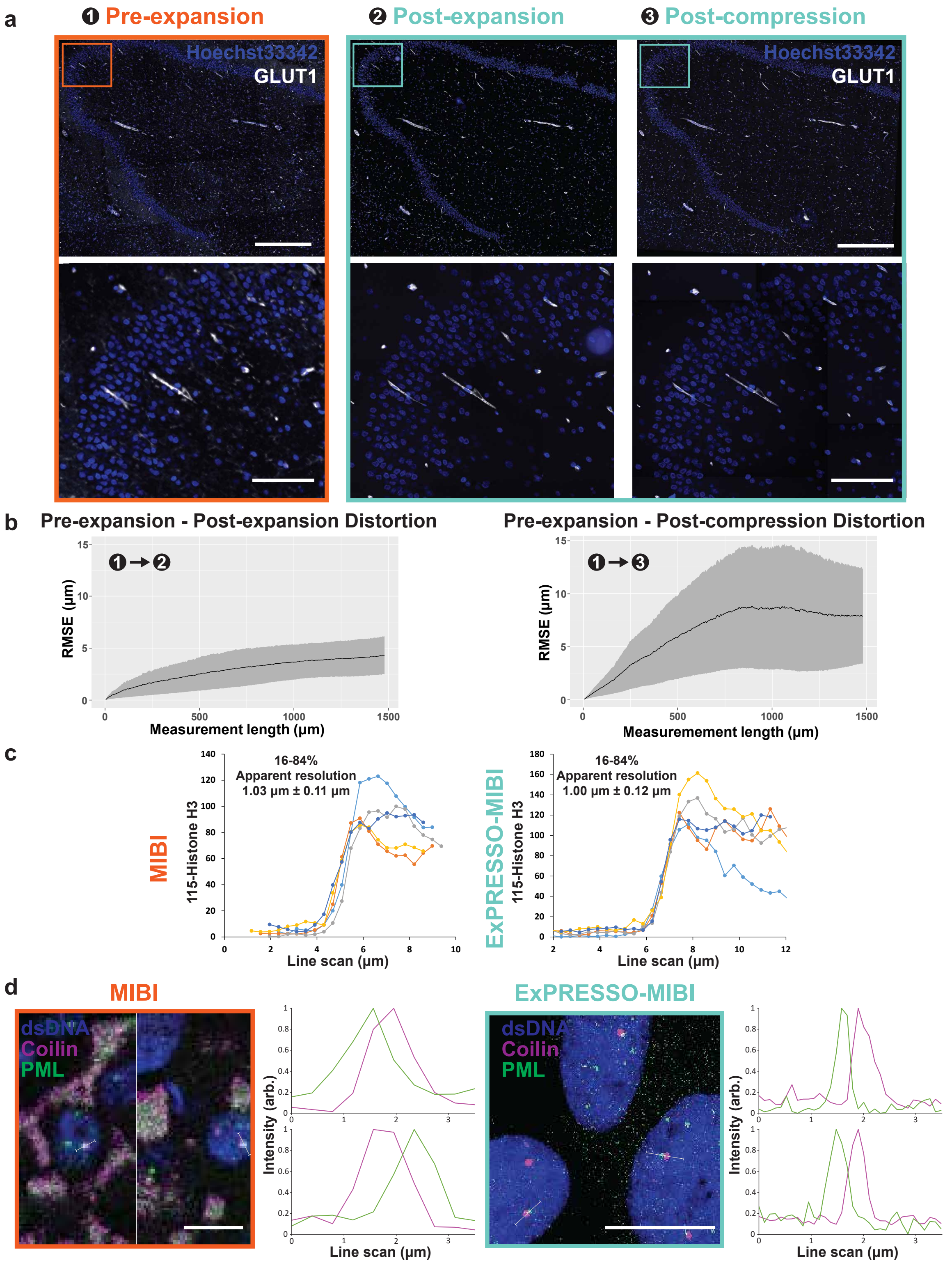

Supplementary Figure 3: Validation of the ExPRESSO workflow, related to Figure 1. **(a)** related to Figure 1c, left: representative views of pre-expansion human FFPE dentate gyrus stained for vessels (GLUT1) and nuclei (Hoechst 33342), with zoom in regions enlarged below. Middle and Right: the same piece of tissue post-expansion and post-compression, respectively, with zoom in regions enlarged below. Scale bar (indicates the pre-expansion dimensions based on a 3.7-fold expansion): 500 µm (top), 100 um (bottom, enlarged). **(b)** Left: Plot showing the root mean square error (RMSE) with plus or minus standard deviation between pre-expansion and post-expansion nuclear channel above. Right: RMSE with plus or minus standard deviation between pre-expansion and post-compression nuclear channel above; Both lengths were back calculated based on a 3.7-fold expansion. **(c)** Apparent resolution of ExPRESSO gels. Line scans were performed on the Histone H3 channel of MIBI (left) and ExPRESSO-MIBI (right) images. The 16–84% criteria resolutions are shown as an average of the five line scans ± s.d (both plots have their x-axis in physical distance). **(d)** Comparison of MIBI and ExPRESSO-MIBI images on HeLa cells stained with dsDNA (Blue), Coilin (Magenta) and PML (Green), with line scans across interacting Cajal and PML bodies. Left: representative MIBI image of pre-expansion HeLa cells, and line scans of an interaction event; Right: representative ExPRESSO-MIBI image of HeLa cells, and line scans of an interaction event; All x-axises for the ExPRESSO-MIBI line scan are back calculated to pre-expansion size based on a 3.7-fold expansion; Scale bar (indicates the pre-expansion dimensions based on a 3.7-fold expansion): 10 µm.

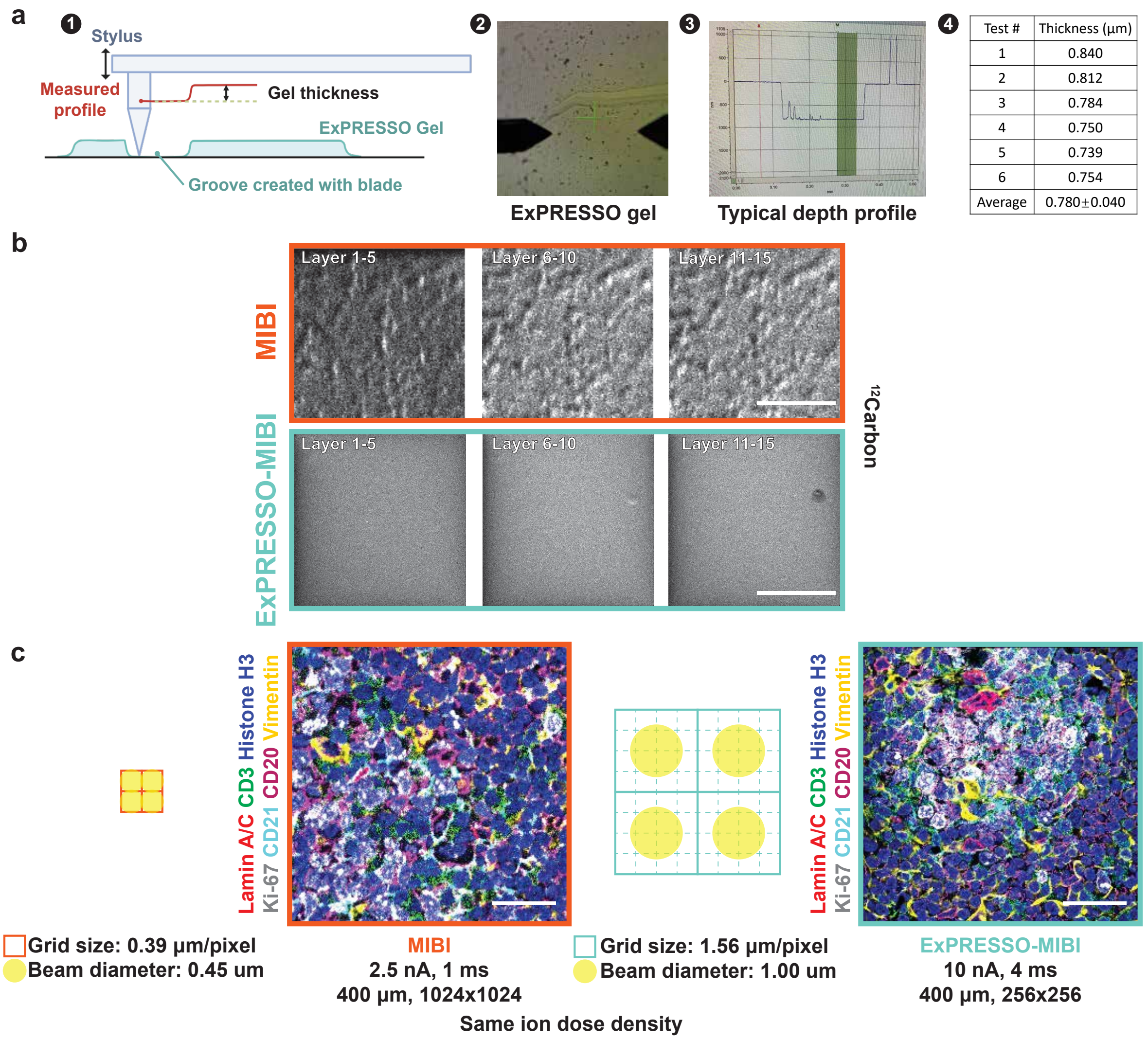

Test #	Thickness (µm)
1	0.840
2	0.812
3	0.784
4	0.750
5	0.739
6	0.754
Average	0.780±0.040

Supplementary Figure 4: Validation of the ExPRESSO workflow, related to Figure 1. (a) Schematic of measurement of thickness of an ExPRESSO gel using a profilometer. (1) Measurements were performed over grooves on the compressed gel created by a razor blade. (2) Representative image of an ExPRESSO gel under the profilometer camera. (3) A representative image of a depth profile. (4) The distance between the gel surface and the valley floor was calculated as the thickness of the gel, thickness data from six measures on the same gel. **(b)** Depth profiling of Human FFPE tonsil sections with MIBI and ExPRESSO-MIBI. Top: Representative ^{12}C MIBI images of a tonsil section, summed every 5 layers. Bottom: Representative ^{12}C MIBI images of an ExPRESSO treated tonsil section, summed every 5 layers. The homogeneous ^{12}C signal distribution at multiple depths indicates the homogeneous nature of the ExPRESSO gels. Scale bars (the ExPRESSO-MIBI scale bars indicate the pre-expansion dimensions based on a 3.7-fold expansion): 50 µm (both). **(c)** Left: Unexpanded tonsil section imaged on MIBI in 2.5 nA “superfine” mode and 400 µm, 1024x1024 resolution, grid size of 0.39 µm/pixel. Right: Parallel-stained ExPRESSO tonsil gel imaged on the MIBI with 10 nA “coarse” mode and 400 µm, 256x256 resolution, grid size of 1.56 µm/pixel (4x larger). These parameters in the ExPRESSO sample match the unexpanded MIBI resolution; there was an increase of beam diameter due to increase of current. This experiment demonstrates that with the same ion dose density for the same size pre-expansion tissue area, the ExPRESSO images had higher counts than the unexpanded images in the Histone H3 channel. The increase of current to match the grid size and beam can produce more ions or save more time. Scale bars (the ExPRESSO-MIBI scale bars indicate the pre-expansion dimensions based on a 3.7-fold expansion): 25 µm.

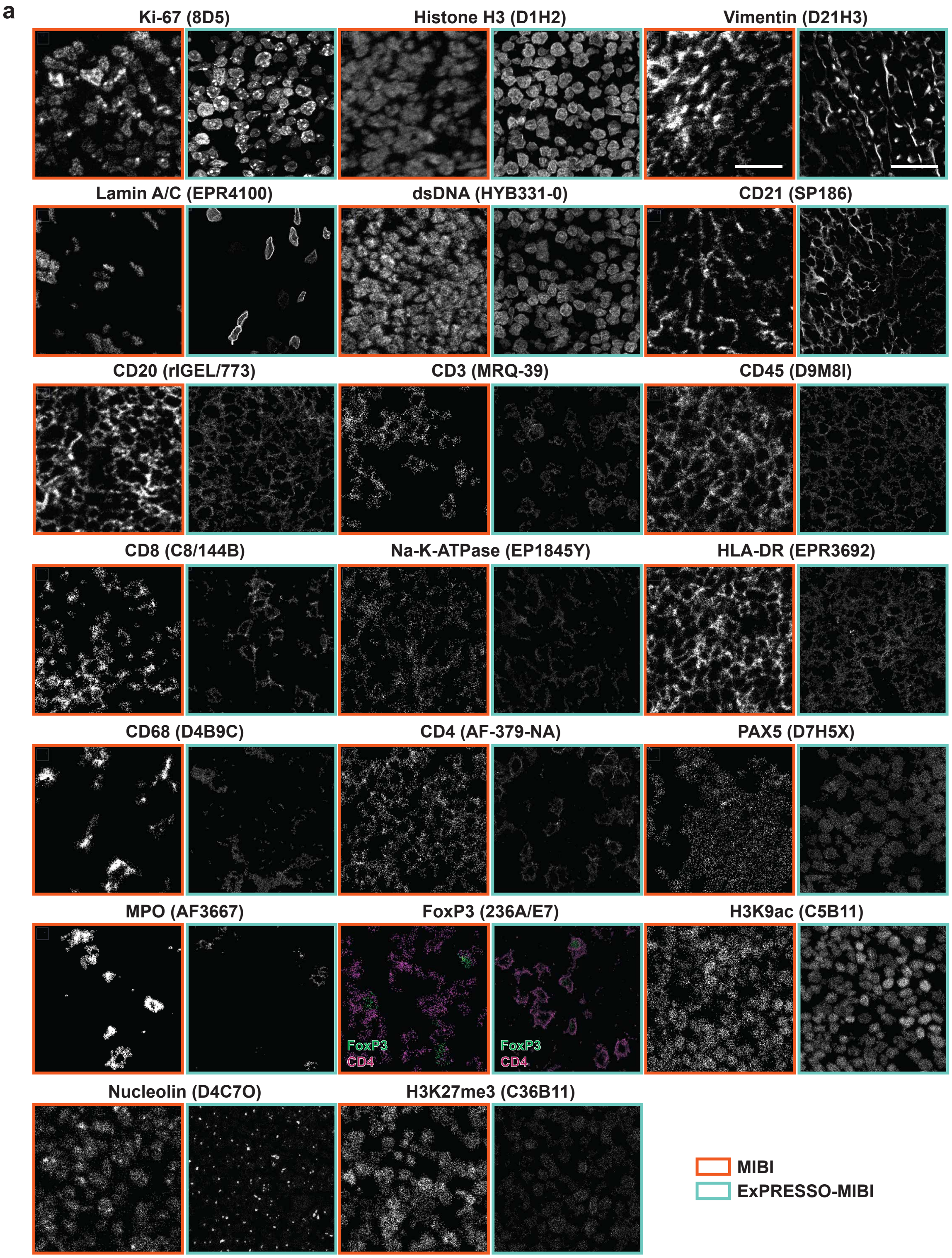

Supplementary Figure 5: Comparison of unexpanded and ExPRESSO imaging in MIBI on human tonsil sections, related to Figure 2. (a) Representative images of human tonsils for each stained marker with clone information in parenthesis in MIBI (left) and ExPRESSO-MIBI (right). Scale bars (the ExPRESSO-MIBI scale bars indicate the pre-expansion dimensions based on a 3.7-fold expansion): 25 µm.

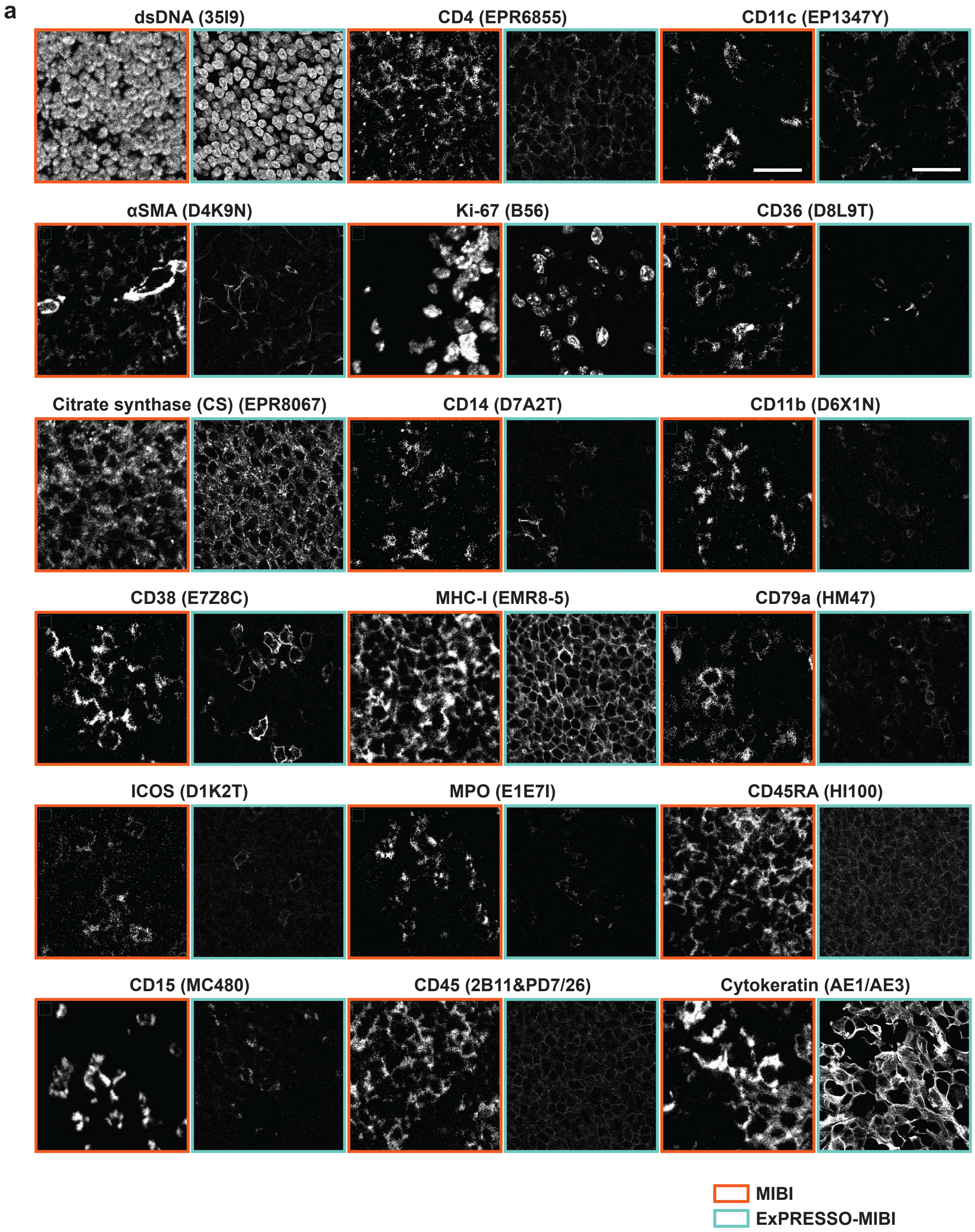

Supplementary Figure 6: Comparison of unexpanded and ExPRESSO imaging in MIBI on human tonsil sections, related to Figure 2. (a) Representative images of human tonsils for each stained marker with clone information in parenthesis in MIBI (left) and ExPRESSO-MIBI (right). Scale bars (the ExPRESSO-MIBI scale bars indicate the pre-expansion dimensions based on a 3.7-fold expansion): 25 μm.

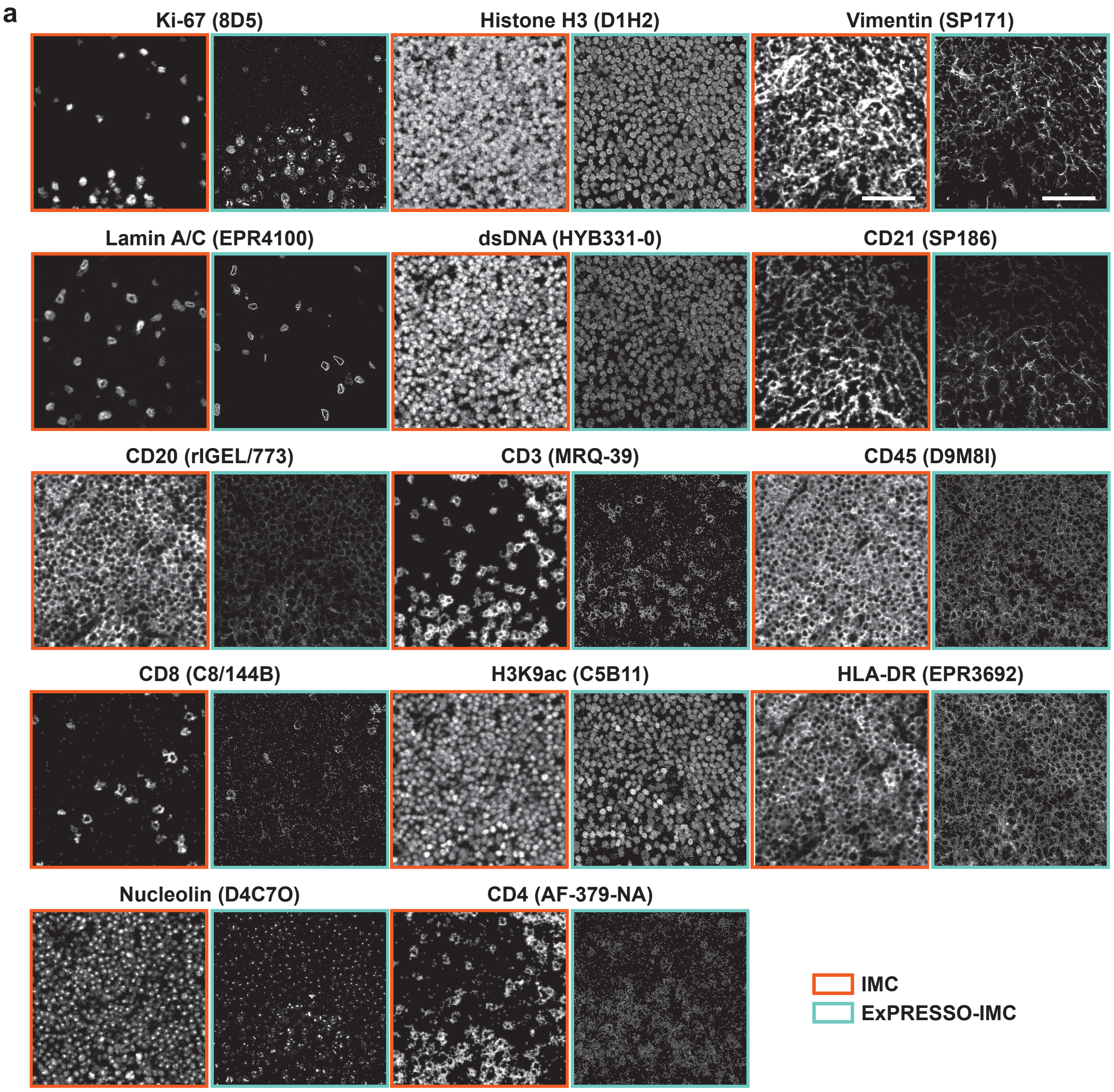

Supplementary Figure 7: Comparison of unexpanded and ExPRESSO imaging in IMC on human tonsil sections, related to Figure 2. (a) Representative images of human tonsils for each stained marker with clone information in parenthesis in IMC (left) and ExPRESSO-IMC (right). Scale bars (the ExPRESSO-IMC scale bars indicate the pre-expansion dimensions based on a 3.7-fold expansion): 50 µm.

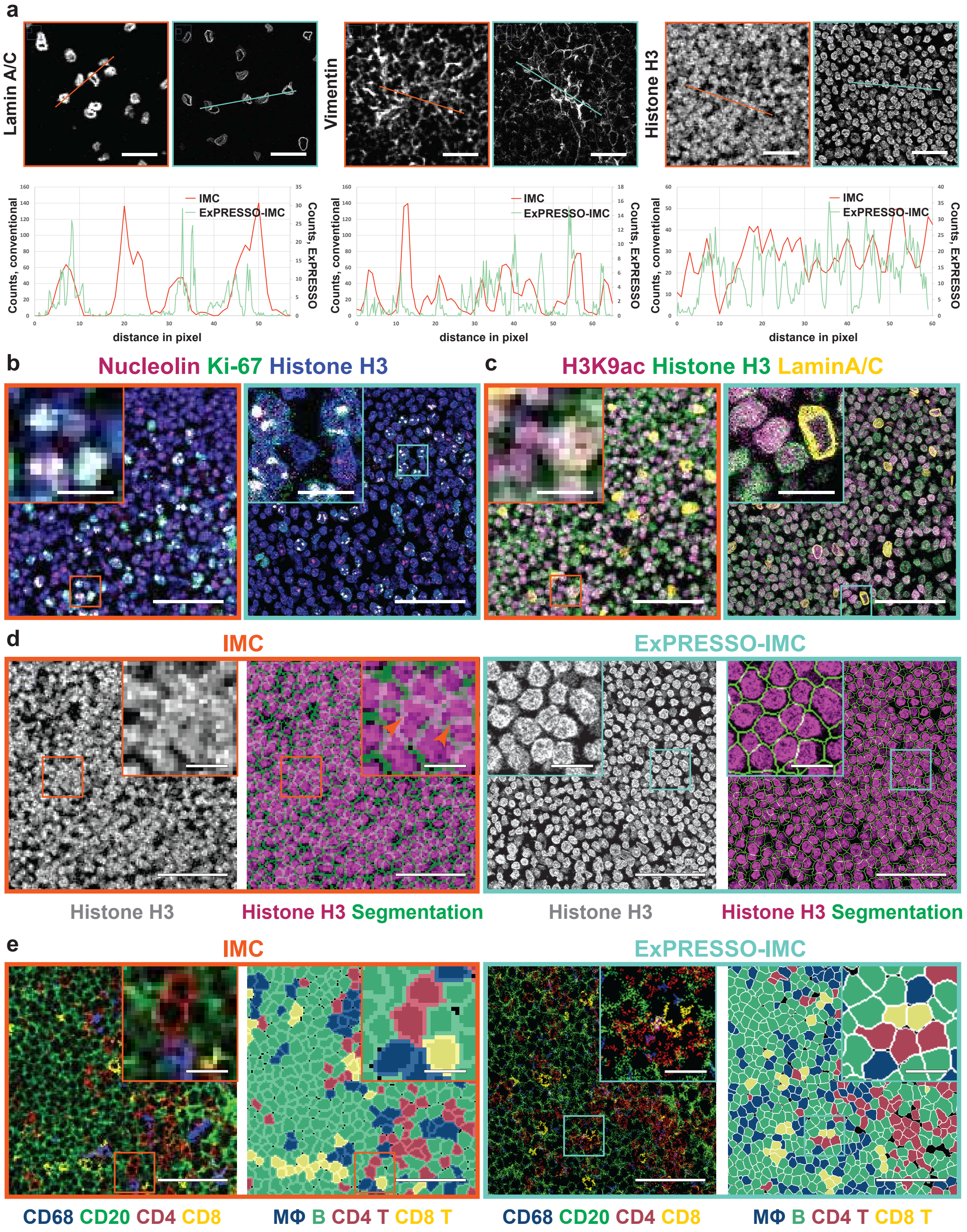

Supplementary Figure 8: ExPRESSO resolves fine details of subcellular structures in IMC on human tonsil sections, related to Figure 3. (a) Upper: Representative IMC images of human tonsils imaged for Lamin A/C (left), Vimentin (middle), and Histone H3 (left). Scale bars (the ExPRESSO-IMC scale bars indicate the pre-expansion dimensions based on a 3.7-fold expansion): 25 µm. Lower: Line scans along the orange (IMC) and cyan (ExPRESSO-IMC) lines in the IMC images. Raw ion counts (Y-axis) were plotted to each individual pixel (X-axis). **(b-c)** Comparison of IMC (left) and ExPRESSO-IMC (right) imaging in human tonsils for (b) resolution of Nucleolin and Ki-67 and (c) histone post-translational modifications revealing spatial positioning of H3K9ac (magenta) and Lamin A/C (yellow). Scale bars (the ExPRESSO-IMC scale bars indicate the pre-expansion dimensions based on a 3.7-fold expansion): 50 µm (main), 10 µm (enlarged). **(d)** Comparison of cell segmentation in IMC (left) and ExPRESSO-IMC (right) imaging in human tonsils. For each group, representative IMC images on the left show nuclei by Histone H3 staining, and images on the right depict a cell segmentation map together with nuclear staining (Histone H3). Scale bars (the ExPRESSO-IMC scale bars indicate the pre-expansion dimensions based on a 3.7-fold expansion): 50 µm (main), 10 µm (enlarged). **(e)** Comparison of cell phenotyping in IMC (left) and ExPRESSO-IMC (right) imaging in human tonsils. For each group, representative IMC images with CD68 (blue), CD20 (green), CD4 (red), and CD8 (yellow), and images on the right depict a cell phenotype map with basic cell type clustered through segmented single cell data. Scale bars (the ExPRESSO-IMC scale bars indicate the pre-expansion dimensions based on a 3.7-fold expansion): 50 µm (main), 10 µm (enlarged).

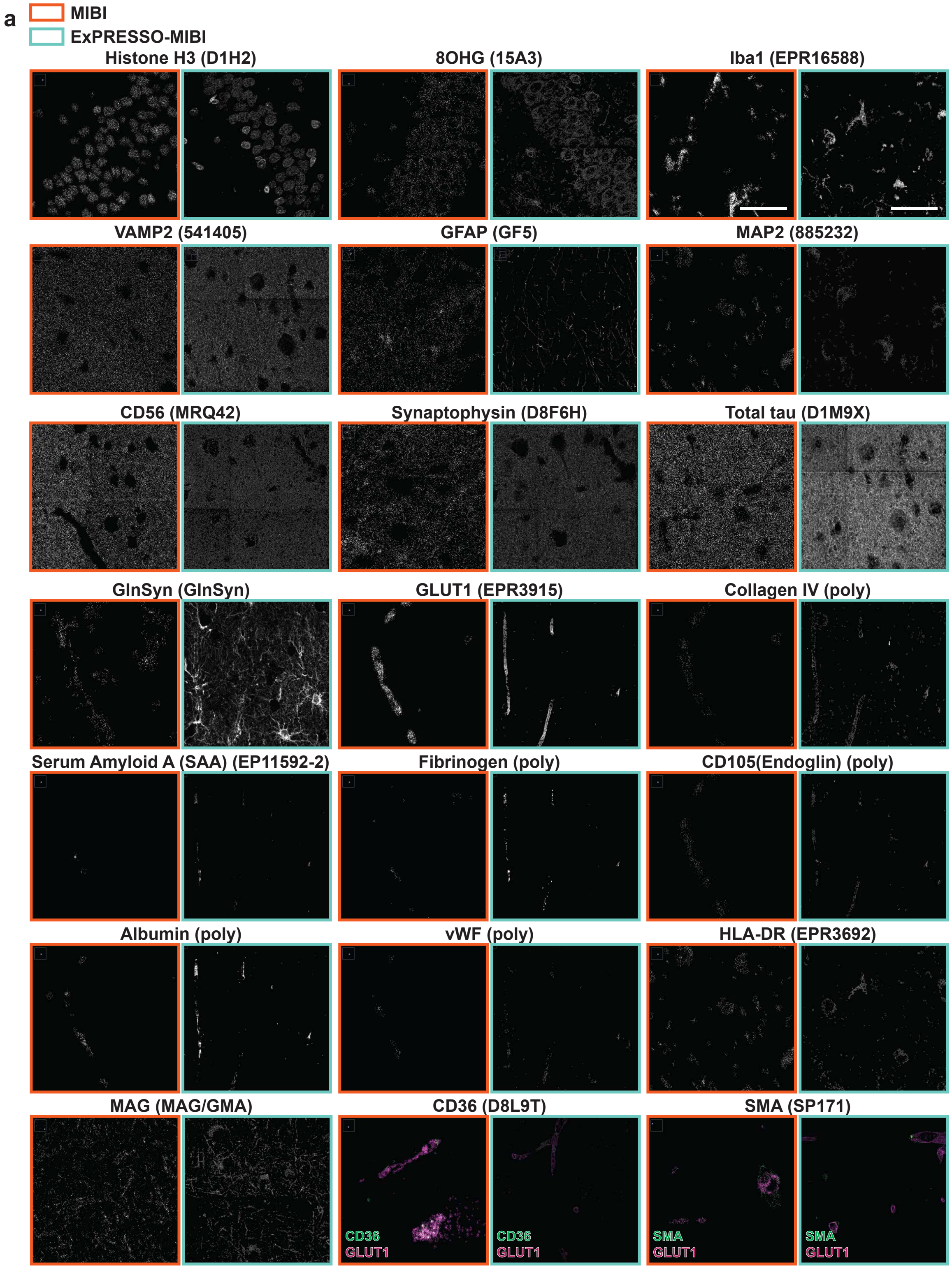

Supplementary Figure 9: ExPRESSO enables high-plex proteomic assessment of the archival human brain sections at subcellular resolution, related to Figure 4. **(a)** Representative images for each stained marker with clone information in parenthesis in MIBI (left in each pair) and ExPRESSO-MIBI (right in each pair) imaging in human brain tissue sections of hippocampus and middle frontal gyrus. Scale bars (the ExPRESSO-MIBI scale bars indicate the pre-expansion dimensions based on a 3.7-fold expansion): 50 µm.

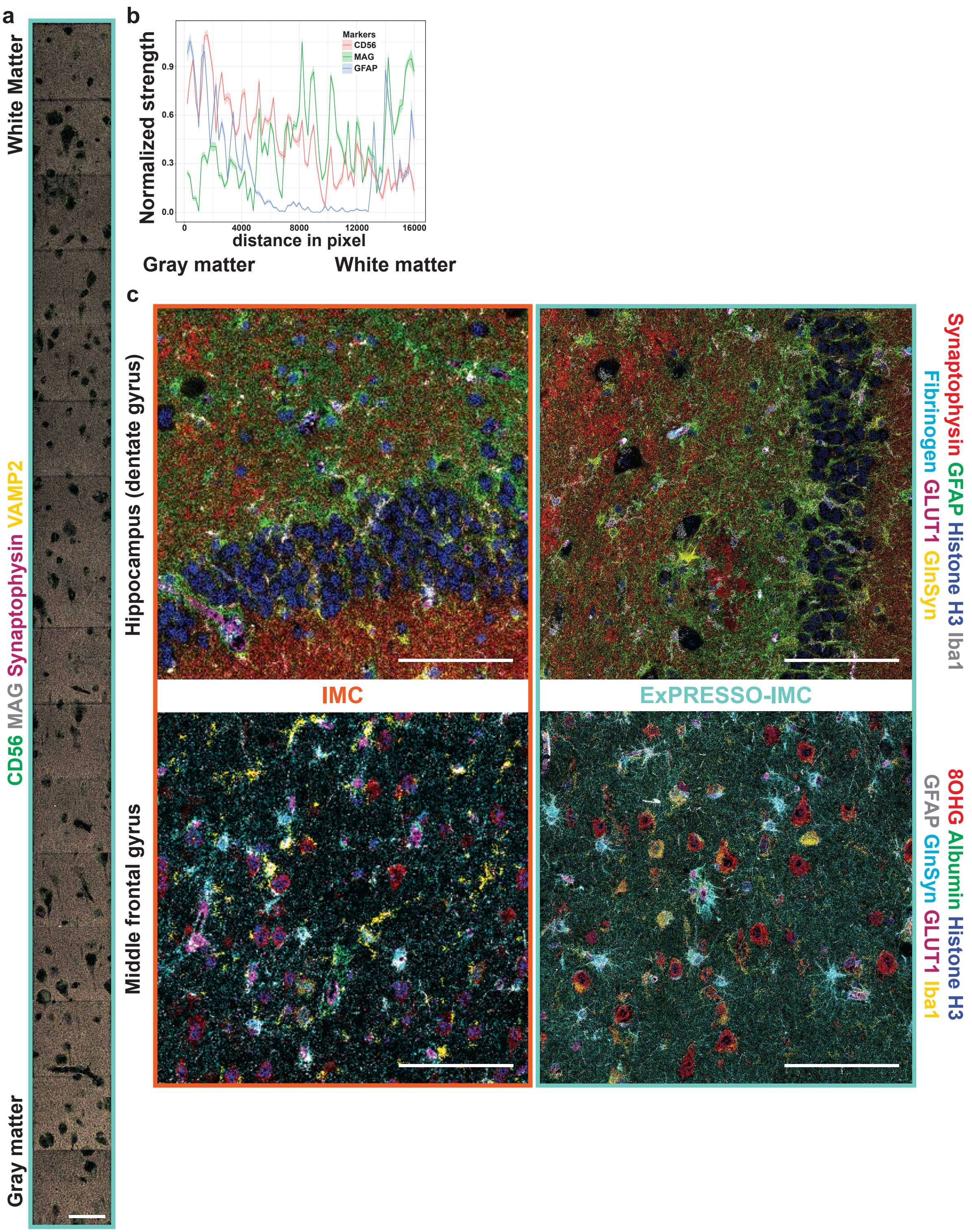

Supplementary Figure 10: ExPRESSO enables high-plex proteomic assessment of the archival human brain sections at subcellular resolution, related to Figure 4. (a) MIBI imaging of an ExPRESSO processed human middle frontal gyrus tissue section, related to Figure 4a. Representative linear acquisition of 16 FOVs, each one with a size of 400x400 µm (physical measurements). Sections were stained for CD56 (neurons), MAG (myelin and oligodendrocytes), and Synaptophysin and VAMP2 (pre-synapse). Scale bars (the ExPRESSO-MIBI scale bars indicate the pre-expansion dimensions based on a 3.7-fold expansion): 50 µm. **(b)** Normalized distributions of three selected channels (CD56, MAG, GFAP) along the transition from gray matter to white matter as shown in Supplementary Figure 10b. The average counts were plotted as solid lines, while plus or minus standard deviation were plotted as a shadow around lines. **(c)** Representative images of IMC and ExPRESSO-IMC of human brain tissue sections from the hippocampus and the middle frontal gyrus. For hippocampus, shown are markers for pre-synapse (Synaptophysin), astrocyte projections (GFAP), nuclei (Histone H3), microglia (Iba1), vessel lumen and BBB leakiness (Fibrinogen), vessels (GLUT1), and muscularized vasculature (SMA). For middle frontal gyrus, shown are markers for neurons with oxidative stress (8OHG), vessel lumen and BBB leakiness (Albumin), astrocytes (GFAP and GlnSyn), vessels (GLUT1), microglia (Iba1), and nuclei (Histone H3). Scale bars (the ExPRESSO-IMC scale bars indicate the pre-expansion dimensions based on a 3.7-fold expansion): 100 µm.

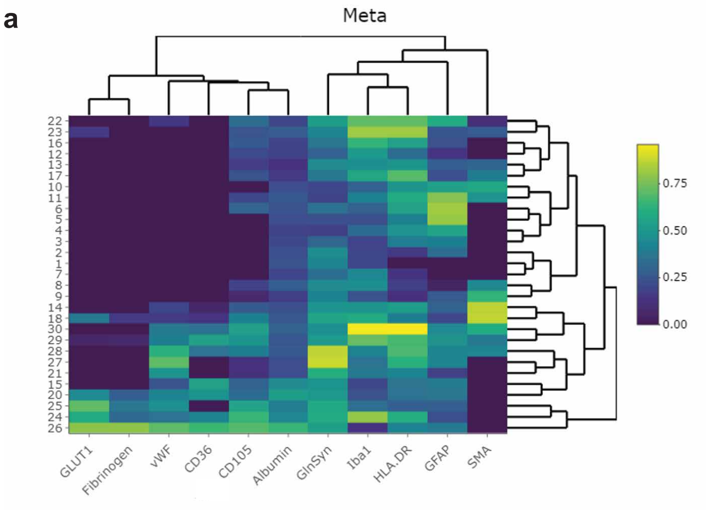

	Cell type	Clustering number
1	Endothelial	18, 20, 25, 26, 15,
2	Astrocytes	28, 27, 21, 14, 7, 8, 9
3	Microglia	16, 17, 22, 23, 24, 29, 30, 13
4	Neurons	1,2,3,4,5,6,10,11,12

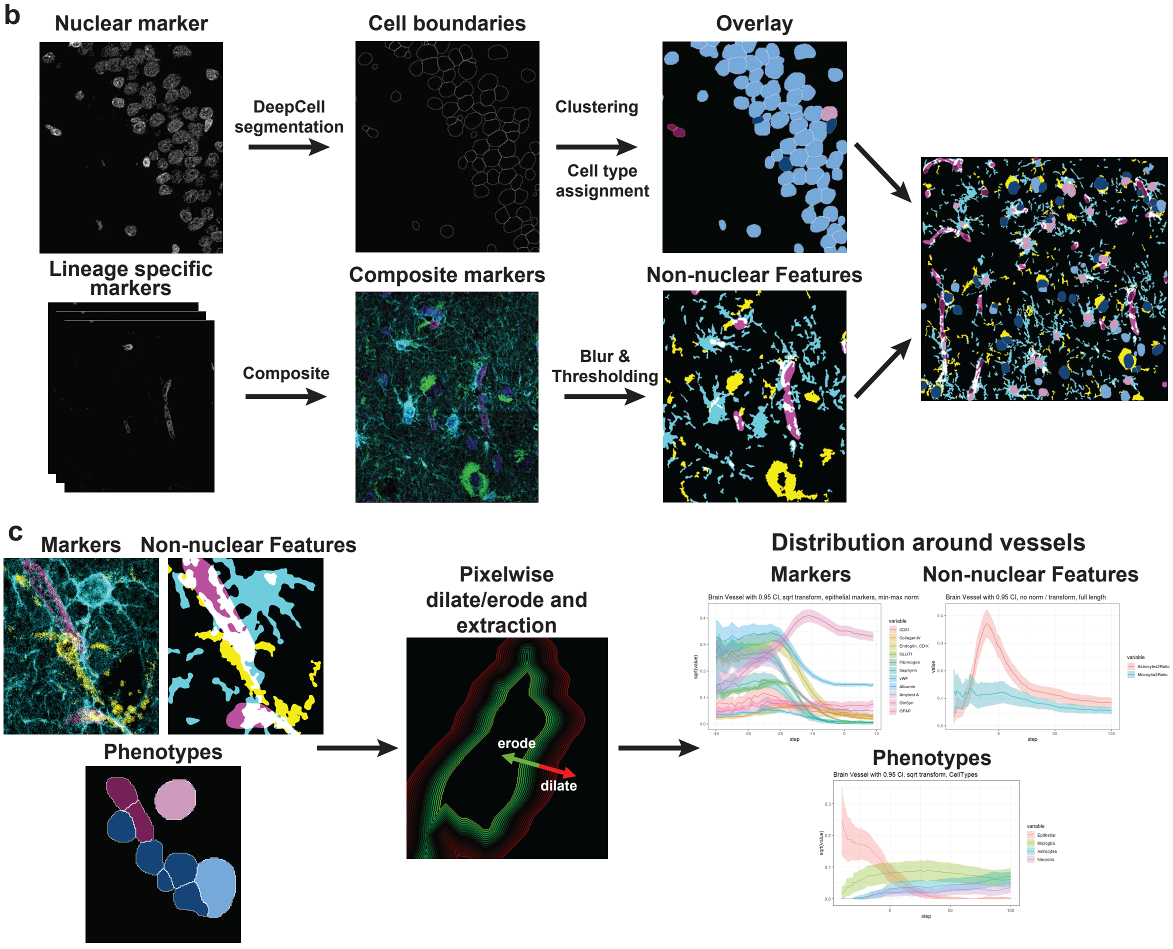

Supplementary Figure 11: Workflow of cell and acellular features extraction on ExPRESSO-MIBI images of human brain sections, related to Figure 5 and 6. (a) Heatmap generated with FlowSOM for the ExPRESSO-MIBI brain sample phenotyping, with each cluster annotation listed on the right. **(b)** Scheme of the pipeline to extract cell phenotyping and non-nuclear features to generate the combinatorial image. **(c)** Scheme of the process of anchoring analysis and pixelwise extraction of markers, features, and cell type information around vessels.

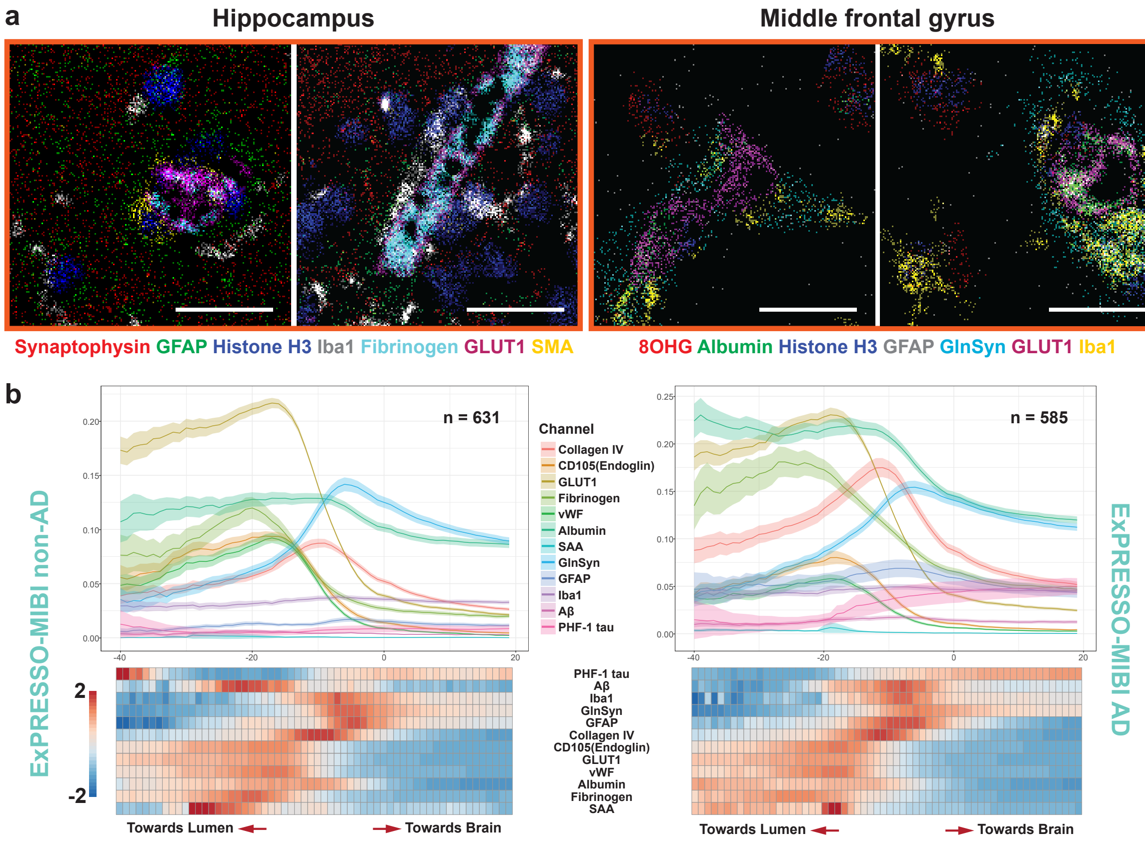

Supplementary Figure 12: Workflow of cell and acellular features extraction on ExPRESSO-MIBI images of human brain sections, related to Figure 5 and 6. (a) Representative MIBI images of non-expanded brain tissues around the BBB, from the hippocampus (left) and the middle frontal gyrus (right). For hippocampus, shown are markers for pre-synapse (Synaptophysin), astrocyte projections (GFAP), nuclei (Histone H3), microglia (Iba1), vessel lumen and BBB leakiness (Fibrinogen), vessels (GLUT1), and muscularized vasculature (SMA). For middle frontal gyrus, shown are markers for neurons with oxidative stress (8OHG), vessel lumen and BBB leakiness (Albumin), astrocytes (GFAP and GlnSyn), vessels (GLUT1), microglia (Iba1), and nuclei (Histone H3). Scale bars: 25 µm. **(b)** Anchoring analysis around vessel feature segment components on ExPRESSO-MIBI images with both non-AD (left) and AD patient samples (right) in both curve and heatmap. The average counts were plotted as solid lines, while the 95% confidence intervals were plotted as a shadow around lines.

Supplementary Note 1

In this manuscript, we primarily focused on ExPRESSO's capability to improve imaging resolution through expansion. Under the same beam size, an ExPRESSO sample will yield better resolution than a conventional sample (Supplementary Figure 13a, Points (1) and (2)). Increased resolution by expansion will also increase acquisition time of the same area as a square of the expansion fold. For instance, a sample with 4x expansion leads to 4^2 = 16x more acquisition time.

In Figure 1h, we explored the possibility of acquiring images in ExPRESSO samples at similar resolution as conventional samples, with the hypothesis that imaging ExPRESSO samples may have advantages in either higher ion yields or faster acquisition times.

With each rastering of the sample surface, the secondary ion yields are proportional to the ion dose delivered to the sample per unit area ([current] × [dwell time] × [pixel number] / [FOV area]). When we apply the same dwell time and calibrate the beam size to fit the expanded pixel size (Supplementary Figure 13a; Points (1) and (3)), the current of the beam increases at a faster rate (Current ∝ d^(8/3), with d as beam size) than the increase in area to be imaged (which increases as the square of the expansion fold). This explains why the increase in beam size will higher yield even though the area to be imaged also increases, since they increase at different rates.

Alternatively, we also reasoned that a decrease in beam dwell time (i.e., the time the beam remains at a given pixel to liberate the lanthanide ions) will allow us to acquire images on ExPRESSO samples with similar ion counts and resolution, but now at a faster speed (since given the same ion dose density, the gain will be [ExM-fold]^(2/3)).

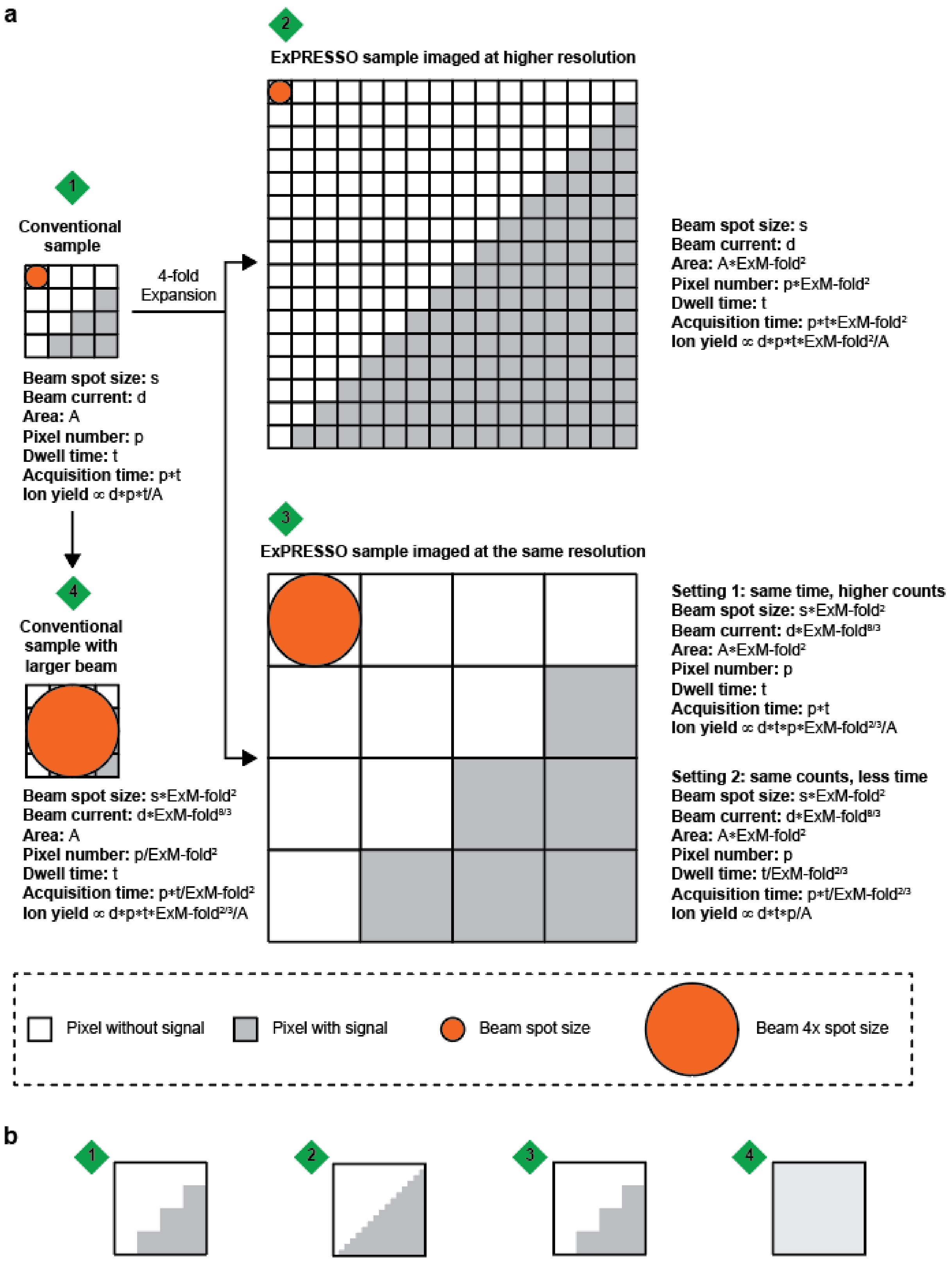
a
1
Conventional sample
4-fold Expansion
Beam spot size: s
Beam current: d
Area: A
Pixel number: p
Dwell time: t
Acquisition time: p*t
Ion yield ∝ d*p*t/A
2
ExPRESSO sample imaged at higher resolution
Beam spot size: s
Beam current: d
Area: A*ExM-fold^2
Pixel number: p*ExM-fold^2
Dwell time: t
Acquisition time: p*t*ExM-fold^2
Ion yield ∝ d*p*t*ExM-fold^2/A
3
ExPRESSO sample imaged at the same resolution
Setting 1: same time, higher counts
Beam spot size: s*ExM-fold^2
Beam current: d*ExM-fold$^{8/3}$
Area: A*ExM-fold^2
Pixel number: p
Dwell time: t
Acquisition time: p*t
Ion yield ∝ d*t*p*ExM-fold$^{2/3}$/A
Setting 2: same counts, less time
Beam spot size: s*ExM-fold^2
Beam current: d*ExM-fold$^{8/3}$
Area: A*ExM-fold^2
Pixel number: p
Dwell time: t/ExM-fold$^{2/3}$
Acquisition time: p*t/ExM-fold$^{2/3}$
Ion yield ∝ d*t*p/A
4
Conventional sample with larger beam
Beam spot size: s*ExM-fold^2
Beam current: d*ExM-fold$^{8/3}$
Area: A
Pixel number: p/ExM-fold^2
Dwell time: t
Acquisition time: p*t/ExM-fold^2
Ion yield ∝ d*p*t*ExM-fold$^{2/3}$/A
Pixel without signal
Pixel with signal
Beam spot size
Beam 4x spot size
b
1
2
3
4

Supplementary Figure 13, (a) scheme of MIBI imaging on unexpanded and ExPRESSO-treated samples, with different resolution and beam size settings. (1) MIBI imaging on a conventional unexpanded sample. Total acquisition time is T_0 = p*t, total ion yield $Y_0 \propto$ d*p*t/A; (2) MIBI imaging on an ExPRESSO sample with expansion factor of 4, but with the same beam size and current. It takes $16T_0$ to finish the acquisition as the pixel numbers increase; (3) MIBI imaging on an ExPRESSO sample with expansion factor of 4, but with beam size (thus resolution) matched with pixel size. Acquisition time is still T_0, total ion yield Y_{ExM} = Y_0*[ExM-fold]^(2/3). Alternatively, the dwell time can be decreased to match the ion yield and thus perform a faster acquisition; (4) acquisition time on a conventional unexpanded sample with the beam size from “3” is decreased under this setting. **(b)** when scaled to original sample size, the signal distribution of above (1) to (4) conditions, shows the difference of resolution under all 4 conditions.

Supplementary Table 1. Paired non-AD&AD samples

Case	AD pathologic Braak stage	AD clinical diagnosis
1	2	not AD
2	2	not AD
3	3	not AD
4	6	AD
5	5	AD
6	4	AD

Note:

Donors were females with age of 66-75, and males with age of 68-75.

Supplementary Table 2. Tissues and antibody panels, Validated antibody for ExPRESSO-MIBI, Tonsil

Specimen	Antibody target	Clone	Provider	Mass channel	Isotope	Titer (μg/mL)	Panel
Tonsil	dsDNA	HYB331-0	Santa Cruz Biotech	89	Y	1.20	1
	Vimentin	D21H3	Abcam	113	In	1.33	1
	Histone H3	D1H2	CST	115	In	1.33	1
	MPO	AF3667	Novus Bio	141	Pr	1.00	1
	CD4	AF-379-NA	Novus Bio	143	Nd	2.00	1
	FoxP3	236A/E7	Abcam	146	Nd	4.00	2
	CD21	SP186	Abcam	150	Nd	0.38	1
	Ki-67	8D5	Novus Bio	151	Eu	1.25	1
	Pax-5	D7H5X	CST	153	Eu	3.00	1
	CD68	D4B9C	CST	156	Gd	2.00	1
	Nucleolin	D4C7O	CST	158	Gd	2.00	2
	CD3	MRQ-39	Cell Marque	159	Tb	1.50	1
	CD20	rIGEL/773	Novus Bio	164	Dy	0.60	1
	LaminA/C	EPR4100	Abcam	165	Ho	1.50	1
	CD8	C8/144B	Cell Marque	166	Er	2.00	1
	HLA-DR	EPR3692	Abcam	167	Er	1.80	1
	CD45	D9M8I	CST	169	Tm	2.00	1
	H3K27me3	C36B11	CST	172	Yb	3.00	2
	H3K9ac	C5B11	CST	174	Yb	2.00	2
	Na-K-ATPase	EP1845Y	Abcam	176	Yb	5.00	1
	dsDNA	35I9 DNA	Abcam	89	Y	1.20	4
	CD4	EPR6855	Ionpath	143	Nd	3.00	4
	CD11c	EP1347Y	Ionpath	144	Nd	2.00	4
	aSMA	D4K9N	CST	147	Sm	2.00	4
	Ki-67	B56	Abcam	151	Eu	1.25	4
	CD36	D8L9T	CST	152	Sm	3.00	4
	CS	EPR8067	Abcm	153	Eu	2.00	4
	CD14	D7A2T	IonPath	154	Sm	3.00	4
	CD11b	D6X1N	IonPath	155	Gd	3.00	4
	CD38	E7Z8C	CST	157	Gd	3.00	4
	MHC-I	EMR8-5	Abcam	158	Gd	2.00	4
	CD79a	HM47	Santa Cruz Biotech	163	Dy	2.00	4
	ICOS	D1K2T	CST	165	Ho	3.00	4
	MPO	E1E7I	IonPath	168	Er	3.00	4
	CD45RA	HI100	BioLegend	171	Yb	2.00	4
	CD15	MC480	BioLegend	173	Yb	1.50	4
	CD45	2B11 & PD7/26	IonPath	175	Lu	3.00	4
	Cytokeratin	AE1/AE3	Abcam	176	Yb	2.00	4
Tested but gave controversial results, or more titration is need in ExPRESSO-MIBI	CD71	MRQ-48	Cell Marque	139	La	3.00	4
	Human cell marker	113-1	Abcam	145	Nd	2.00	4
	PD-1	D4W2J	IonPath	148	Nd	3.00	4
	CD31	EPR17259	Abcam	150	Nd	3.00	4
	CD45RO	UCH-L1	BioLegend	169	Tm	2.00	4
	CD138	SP152	IonPath	174	Yb	3.00	4
	Granzyme B	EPR20129-217	Abcam	148	Nd	1.50	2
	CD163	EDHu-1	Novus Bio	155	Gd	1.50	2
	CD11b	EPR1344	Abcam	160	Gd	2.00	2

Note:
*When placed side by side, the conventional and ExPRESSO MIBI/IMC samples were stained by the exact same batch of antibody cocktails

Supplementary Table 3. Tissues and antibody panels, Validated antibody for ExPRESSO-MIBI, Brain

Specimen	Antibody target	Clone	Provider	Mass channel	Isotope	Titer (µg/mL)	Panel
Hippocampus & Middle and frontal Gyrus	Histone H3	D1H2	CST	89	Y	2.00	2
	SMA	SP171	Abcam	113	In	0.75	3
	Total Tau	D1M9X	CST	115	In	0.75	3
	MAP2	885232	R&D	141	Pr	3.00	2
	CD56	MRQ42	Cell Marque	145	Nd	1.80	3
	GlnSyn	GlnSyn	Abcam	151	Eu	1.50	2
	Iba1	EPR16588	Abcam	152	Sm	1.50	2
	CollagenIV	Polyclonal	Abcam	154	Sm	1.50	2
	Albumin	Polyclonal	Sigma	156	Gd	1.50	2
	CD36	D8L9T	CST	157	Gd	3.00	3
	CD105(Endoglin)	Polyclonal	R&D	158	Gd	1.50	2
	Amyloid A	EP11592-92	Abcam	160	Gd	3.00	2
	VAMP2	541405	R&D	161	Dy	0.75	2
	GLUT1	EPR3915	Abcam	162	Dy	3.00	2
	vWF	Polyclonal	Abcam	163	Dy	1.50	2
	MAG	MAG/GMA	Abcam	165	Ho	3.00	3
	Synaptophysin	D8F6H	CST	168	Er	3.00	2
	Fibrinogen	Polyclonal	Dako	171	Yb	1.50	2
	GFAP	GF5	Invitrogen	174	Yb	1.50	2
	8OHG	15A3	Abcam	175	Lu	3.00	2
	HLA-DR	EPR3692	Abcam	176	Yb	1.50	3
Validated in AD samples	Ab(pan)	4G8	Biolegend	175	Yb	0.75	5
	PHF1-TAU	PHF-1	available through Dr. Peter Davies	176	Yb	0.75	5
Tested but gave controversial results in ExPRESSO-MIBI	CD31	EP3095	Abcam	148	Nd	3.00	3
	VGLUT1	N28-9	Abcam	166	Er	5.00	3
	VGLUT2	8G9.2	Abcam	167	Er	5.00	3
	PSD95	Polyclonal	Abcam	170	Er	3.00	3
	Gephyrin	G-6	Santa Cruz	172	Yb	1.25	3
	CD44	156-3611	Invitrogen	173	Yb	1.50	3

Note:

*When placed side by side, the conventional and ExPRESSO MIBI/IMC samples were stained by the exact same batch of antibody cocktails

Supplementary Table 4. Tissues and antibody panels, Validated antibody for ExPRESSO-MIBI, HeLa cells

Specimen	Antibody target	Clone	Provider	Mass channel	Isotope	Titer (µg/mL)	Panel
HeLa cells	dsDNA	35I9DNA	Abcam	89	Y	2.00	6
	Coilin	IH10	Abcam	145	Nd	5.00	6
	Ki-67	B56	Abcam	151	Eu	0.25	6
	LaminA/C	EPR4100	Abcam	165	Ho	1.00	6
	Fibrillarin	C13C3	CST	167	Er	1.25	6
	PML	E6S9L	CST	172	Yb	4.00	6

Note:

*When placed side by side, the conventional and ExPRESSO MIBI/IMC samples were stained by the exact same batch of antibody cocktails

Supplementary Table 5. Tissues and antibody panels, Validated antibody for ExPRESSO-IMC, Tonsil

Specimen	Antibody target	Clone	Provider	Mass channel	Isotope	Titer (µg/mL)	Panel
Tonsil	dsDNA	HYB331-0	Santa Cruz Biotech	89	Y	1.20	3
	Vimentin	SP171	Abcam	113	In	1.33	3
	Histone H3	D1H2	CST	115	In	1.33	3
	CD4	AF-379-NA	Novus Bio	143	Nd	2.00	3
	CD21	SP186	Abcam	150	Nd	0.38	3
	Ki-67	8D5	Novus Bio	151	Eu	1.25	3
	Nucleolin	D4C7O	CST	158	Gd	2.00	3
	CD3	MRQ-39	Cell Marque	159	Tb	1.50	3
	CD20	rIGEL/773	Novus Bio	164	Dy	0.60	3
	LaminA/C	EPR4100	Abcam	165	Ho	1.50	3
	CD8	C8/144B	Cell Marque	166	Er	2.00	3
	HLA-DR	EPR3692	Abcam	167	Er	1.80	3
	CD45	D9M8I	CST	169	Tm	2.00	3
	H3K9ac	C5B11	CST	174	Yb	2.00	3

Note:

*When placed side by side, the conventional and ExPRESSO MIBI/IMC samples were stained by the exact same batch of antibody cocktails

Supplementary Table 6. Tissues and antibody panels, Validated antibody for ExPRESSO-IMC, Brain

Specimen	Antibody target	Clone	Provider	Mass channel	Isotope	Titer (µg/mL)	Panel
Hippocampus & Middle and frontal Gyrus	Histone H3	D1H2	CST	89	Y	2.00	2
	SMA	SP171	Abcam	113	In	0.75	3
	Total Tau	D1M9X	CST	115	In	0.75	3
	MAP2	885232	R&D	141	Pr	3.00	2
	CD56	MRQ42	Cell Marque	145	Nd	1.80	3
	GlnSyn	GlnSyn	Abcam	151	Eu	1.50	2
	Iba1	EPR16588	Abcam	152	Sm	1.50	2
	CollagenIV	Polyclonal	Abcam	154	Sm	1.50	2
	Albumin	Polyclonal	Sigma	156	Gd	1.50	2
	CD105(Endoglin)	Polyclonal	R&D	158	Gd	1.50	2
	Amyloid A	EP11592-92	Abcam	160	Gd	3.00	2
	VAMP2	541405	R&D	161	Dy	0.75	2
	GLUT1	EPR3915	Abcam	162	Dy	3.00	2
	vWF	Polyclonal	Abcam	163	Dy	1.50	2
	Synaptophysin	D8F6H	CST	168	Er	3.00	2
	Fibrinogen	Polyclonal	Dako	171	Yb	1.50	2
	GFAP	GF5	Invitrogen	174	Yb	1.50	2
	8OHG	15A3	Abcam	175	Lu	3.00	2
	HLA-DR	EPR3692	Abcam	176	Yb	1.50	3
no signal	CD31	EP3095	Abcam	148	Nd	3.00	3

Note:

*When placed side by side, the conventional and ExPRESSO MIBI/IMC samples were stained by the exact same batch of antibody cocktails

Supplementary Table 7. Imaging conditions of MIBI/ExPRESSO-MIBI experiments

Figure	Current (nA)	FOV size (µm)*	dwell time (ms)	planes	pixels	Ion dose/plane (nA×hr/mm2)	Ion dose total	Time per FOV (min)	Source/Machine
1d, f	5	400	1	1	1024	9.102	9.102	17.5	Betty
1f (bottom)	5	400	1	1	1024	9.102	9.102	17.5	Betty
1g (top)	2.5/5/10	400	1	1	512	1.138/2.276/4.551	1.138/2.276/4.551	4.5	Production
1h (bottom left, MIBI)	2.5	400	1	1	1024	4.551	4.551	17.5	Production
1h (bottom right, ExPRESSO-MIBI)	10	400	4	1	256	4.551	4.551	17.5	Production
2a	5	400	1	1	1024	9.102	9.102	17.5	Betty
3a, c	5	400	1	1	1024	9.102	9.102	17.5	Betty
3b	5	400	1	1	1024	9.102	9.102	17.5	Production
4a, c	5	400	1	2	1024	9.102	18.204	35.0	Betty
4b	3.6	400	5	3	512	8.192	24.576	65.6	Alpha
5a, b, c	5	400	1	2	1024	9.102	18.204	35.0	Betty
6a, b	5	400	1	1	1024	9.102	9.102	17.5	Production
S1d	5	600/2000	0.008	N/A	512	N/A	N/A	N/A	Betty**
S1e (left)	3.6	400	5	3	512	8.192	24.576	65.6	Alpha
S1e (right)	5	400	1	1	1024	9.102	9.102	17.5	Production
S1f (left)	5	1658	0.01	N/A	512	N/A	N/A	N/A	Production**
S1k (Left)	5	200	2	1	512	18.204	18.204	8.5	Production
S1k (Right)	5	400	1	1	1024	9.102	9.102	17.5	Production
S1m	3.6	400	1	15	512	1.638	24.576	65.6	Alpha
S1n (left, MIBI)	2.5	400	1	1	1024	4.551	4.551	17.5	Production
S1n (right, ExPRESSO-MIBI)	10	400	4	1	256	4.551	4.551	17.5	Production
S2a	5	400	1	1	1024	9.102	9.102	17.5	Betty
S2a-continue	5	400	1	1	1024	9.102	9.102	17.5	Production
S4a, b	5	400	1	2	1024	9.102	18.204	35.0	Betty
S5d	5	400	1	2	1024	9.102	18.204	35.0	Betty
S5e	5	400	1	1	1024	9.102	9.102	17.5	Production

Note:

Alpha: a custom-build alpha-iteration MIBI-TOF mass spectrometer equipped with a duoplasmatron ion gun (Ionpath) which emits O_2^+

Betty: a custom-build beta-iteration MIBI-TOF mass spectrometer equipped with a Hyperion™ ion source (Oregon Physics) that generates Xe^+

Production: commercially available MIBI-TOF mass spectrometer (MIBIscope™ System) produced by IonPath, Inc, equipped with a Hyperion™ ion source (Oregon Physics) that generates Xe^+

*The size of the FOVs in one MIBI/IMC run, the presented images in Figures can be stitched or cropped

**To acquire SED image, no real run executed

Supplementary Table 8. Imaging conditions of IMC/ExPRESSO-IMC experiments

Figure	Energy level (dB)	FOV size (µm)*	Time per FOV (min)**	Source/Machine
2b (left, middle, IMC)	1	800x800	~64.0	IMC
2b (right, ExPRESSO-IMC)	0	1200x1200	~144.0	IMC
S2b (left, IMC)	1	800x800	~64.0	IMC
S2b (right, ExPRESSO-IMC)	0	1200x1200	~144.0	IMC
S3a, b, c (left, IMC)	1	800x800	~64.0	IMC
S3a, b, c (right, ExPRESSO-IMC)	0	1200x1200	~144.0	IMC
S4d (left, IMC)	1	400x400	~16.0	IMC
S4d (right, ExPRESSO-IMC)	0	1200x1200	~144.0	IMC

Note:

IMC: a Hyperion Imaging Mass Cytometry (IMC, Fluidigm) with a laser ablation source

*The size of the FOVs in one MIBI/IMC run, the presented images in Figures can be stitched or cropped

**The pixel size for IMC is 1 µm/pixel, and a rough estimation of IMC acquisition speed is 10000 pixel/min

Supplementary Table 9. Segmentation parameters

Methods	Tissue type	model_mpp*	Nuclear channel	Membrane channel
ExPRESSO MIBI	Tonsil	0.25 (0.6.0)	Histone H3	CD45
Conventional MIBI	Tonsil	0.6 (0.9.1)	Histone H3	CD45
ExPRESSO MIBI	Brain	0.1 (0.9.1)	Histone H3	All zero dummy image
ExPRESSO IMC	Tonsil	0.8 (0.6.0)	Histone H3	CD45
Conventional IMC	Tonsil	2.0 (0.6.0)	Histone H3	CD45

Note:

*deepcell-tf version and their weight file address

0.6.0: https://deepcell-data.s3-us-west-1.amazonaws.com/model-weights/Multiplex_Segmentation_20200908_2_head.h5.

0.9.1: https://deepcell-data.s3-us-west-1.amazonaws.com/saved-models/MultiplexSegmentation-7.tar.gz

Supplementary Table 10. Signals comparison

	Non (counts/pixel)	ExPRESSO (counts/pixel)	Ratio* (Non/ExPRESSO, expected 13.7**)
Histone H3	33.04	58.65	0.56
Vimentin	11.77	17.14	0.69
dsDNA	7.15	24.69	0.29
CD20	5.16	0.68	7.54
HLA-DR	2.55	0.69	3.71
MHC-I	2.28	1.78	1.28
CS	1.91	4.43	0.43
CD45RA	1.88	0.39	4.8
CD45	1.26	0.22	5.75
Ki-67	0.75	0.47	1.59
CD79a	0.65	0.15	4.22
Cytokeratin	0.3	2.39	0.12
CD4	0.21	0.43	0.49
CD11b	0.19	0.02	8.15
CD3	0.16	0.24	0.67
CD38	0.16	0.23	0.69
CD11c	0.1	0.05	2.15

Note:

*Base on adjacent non-expand and ExPRESSO Tonsil sections with roughly the same region, stained with the same batch of antibody cocktail, imaged with the same machine with the same settings; Both have pixel size of ~400 nm, the total area acquired on the ExPRESSO sample is [ExM fold]^2 fold of the non-expand one.

**If consider the expansion fold (3.7x), the per pixel counts from non-expand sample should be [ExM fold]^2 higher
