# Supplementary material for: Expanded vacuum-stable gels for multiplexed high-resolution spatial histopathology
Source: Nat Commun. 2023 Jul 7;14:4013. doi: 10.1038/s41467-023-39616-w (PMC10329015; doi:10.1038/s41467-023-39616-w)
Supplement: Supplementary file 3 — Reporting Summary [file 41467_2023_39616_MOESM3_ESM.pdf]

nature research

Corresponding author(s): Xavier Rovira-Clavé, Garry P. Nolan, Sizun Jiang

Last updated by author(s): May 21, 2023

# Reporting Summary

Nature Research wishes to improve the reproducibility of the work that we publish. This form provides structure for consistency and transparency in reporting. For further information on Nature Research policies, see our Editorial Policies and the Editorial Policy Checklist.

## Statistics

For all statistical analyses, confirm that the following items are present in the figure legend, table legend, main text, or Methods section.

| n/a | Confirmed | |
|---|---|---|
| ☐ | ☒ | The exact sample size (*n*) for each experimental group/condition, given as a discrete number and unit of measurement |
| ☒ | ☐ | A statement on whether measurements were taken from distinct samples or whether the same sample was measured repeatedly |
| ☒ | ☐ | The statistical test(s) used AND whether they are one- or two-sided<br>*Only common tests should be described solely by name; describe more complex techniques in the Methods section.* |
| ☒ | ☐ | A description of all covariates tested |
| ☒ | ☐ | A description of any assumptions or corrections, such as tests of normality and adjustment for multiple comparisons |
| ☐ | ☒ | A full description of the statistical parameters including central tendency (e.g. means) or other basic estimates (e.g. regression coefficient) AND variation (e.g. standard deviation) or associated estimates of uncertainty (e.g. confidence intervals) |
| ☒ | ☐ | For null hypothesis testing, the test statistic (e.g. *F*, *t*, *r*) with confidence intervals, effect sizes, degrees of freedom and *P* value noted<br>*Give P values as exact values whenever suitable.* |
| ☒ | ☐ | For Bayesian analysis, information on the choice of priors and Markov chain Monte Carlo settings |
| ☒ | ☐ | For hierarchical and complex designs, identification of the appropriate level for tests and full reporting of outcomes |
| ☒ | ☐ | Estimates of effect sizes (e.g. Cohen's *d*, Pearson's *r*), indicating how they were calculated |

*Our web collection on statistics for biologists contains articles on many of the points above.*

## Software and code

Policy information about availability of computer code

| | |
|---|---|
| Data collection | MIBI acquisition was performed on a commercially available MIBIscope System from Ionpath with MIBIcontrol v.1.7.0-0f60ffbc, MIBI image processing was performed using MIBI Analysis tools (https://github.com/lkeren/MIBIAnalysis) and the toffy package (https://github.com/angelolab/toffy) along with minimal custom codes. MCD files were converted to single-marker tiff images using a custom Python script developed by the Bodenmiller group (https://github.com/BodenmillerGroup/imctools). For single cell data clustering, R package of FlowSOM (2.4.0) was used for clustering of Brain cells, while R package of Seurat (3.2.3) was used to cluster multiplexed data of MIBI/IMC on Tonsil. |
| Data analysis | The codes for anchoring analysis are made available at https://github.com/yunhaoBai/Anchoring_analysis, which also has been deposited in the Zenodo under accession code DOI: 10.5281/zenodo.7949476. |

For manuscripts utilizing custom algorithms or software that are central to the research but not yet described in published literature, software must be made available to editors and reviewers. We strongly encourage code deposition in a community repository (e.g. GitHub). See the Nature Research guidelines for submitting code & software for further information.

## Data

Policy information about availability of data

All manuscripts must include a data availability statement. This statement should provide the following information, where applicable:

- Accession codes, unique identifiers, or web links for publicly available datasets
- A list of figures that have associated raw data
- A description of any restrictions on data availability

All the multiplexed imaging data (MIBI and IMC), and other images used in Figures of this work, have been deposited in the Zenodo under accession code DOI:10.5281/zenodo.7960511.

# Field-specific reporting

Please select the one below that is the best fit for your research. If you are not sure, read the appropriate sections before making your selection.

☒ Life sciences ☐ Behavioural & social sciences ☐ Ecological, evolutionary & environmental sciences

For a reference copy of the document with all sections, see nature.com/documents/nr-reporting-summary-flat.pdf

# Life sciences study design

All studies must disclose on these points even when the disclosure is negative.

| | |
|---|---|
| Sample size | No sample size calculation was performed because this manuscript focuses on demonstration of ExPRESSO methodology development.<br>All the multiplexed tonsil images were acquired on multiple sections from the same donor, including both MIBI and IMC. The brain sections were from paired Alzheimer's Disease (AD) and non-AD patients, with information reported in Supplementary Information, Supplementary Table 1. |
| Data exclusions | No data were excluded from the analyses. |
| Replication | The replication of the ExPRESSO methodology was verified in several tissue types (tonsil, brain), imaging modalities (MIBI, IMC), and imaging conditions (AD vs non-AD, different machine parameters).<br>Antibody staining: Once an antibody is titrated and validated, its staining is stable in replicated experiments.<br>Gel processing: Specifically, in the revision we processed 12 sections from 6 patients (each one has two from Hippocampus and middle frontal gyrus, respectively), all were successfully expanded, stained, compressed, and imaged (DOI:10.5281/zenodo.7960511., MIBI_MFG-HIP_ExPRESSO_Production_400um_1024x1024_1ms_1depth_all.zip). |
| Randomization | Randomization was not necessary for this study. Randomization is not relevant for this study because the experiments were aiming to validate technical performance. |
| Blinding | Blinding was not necessary for this study. Blinding is not relevant for this study because the experiments were aiming to validate technical performance. |

# Reporting for specific materials, systems and methods

We require information from authors about some types of materials, experimental systems and methods used in many studies. Here, indicate whether each material, system or method listed is relevant to your study. If you are not sure if a list item applies to your research, read the appropriate section before selecting a response.

## Materials & experimental systems

| n/a | Involved in the study | |
|---|---|---|
| ☐ | ☒ | Antibodies |
| ☐ | ☒ | Eukaryotic cell lines |
| ☒ | ☐ | Palaeontology and archaeology |
| ☒ | ☐ | Animals and other organisms |
| ☒ | ☐ | Human research participants |
| ☒ | ☐ | Clinical data |
| ☒ | ☐ | Dual use research of concern |

## Methods

| n/a | Involved in the study | |
|---|---|---|
| ☒ | ☐ | ChIP-seq |
| ☒ | ☐ | Flow cytometry |
| ☒ | ☐ | MRI-based neuroimaging |

## Antibodies

| | |
|---|---|
| Antibodies used | All clones, titers, vendors, catalog numbers, and conjugation information can be found in Supplementary Data, Table S2. Tissues and antibodies. |
| Validation | All multiplexed staining images were compared to online databases, including the Human Protein Atlas, and multiplexed images (MIBI) acquired in previous publications on similar systems. They are then reviewed by at least 3 separate individuals, including a pathologist or subject matter expert for consensus-based quality control.<br>All antibody clones used in this study have also been cross validated with the staining pattern and associated IHC image pattern in previous published multiplexed imaging studies by our laboratories (Vijayaragavan, K., et al., Acta Neuropathologica Communications, 2022; Jiang, S.Z., et al., Immunity, 2022). |

## Eukaryotic cell lines

Policy information about cell lines

| | |
|---|---|
| Cell line source(s) | HeLa (ATCC) |
| Authentication | None of the cell lines used were authenticated. |
| Mycoplasma contamination | All cell lines were tested negative for Mycoplasma contamination. |
| Commonly misidentified lines (See ICLAC register) | No commonly misidentified cell lines were used in this study. |
